# Internet use for health information, health service utilization, and quality of care in the U.S.

**DOI:** 10.1186/s12913-025-12807-5

**Published:** 2025-05-08

**Authors:** Diana Alma Taya, Ying-Chih Chuang

**Affiliations:** School of Public Health, Taipei Medical University, 235 10th Floor, Biomedical Technology Building, No. 301, Yuantong Road, Zhonghe District, New Taipei City, Taiwan

**Keywords:** Quality of health care, Patient-Centered care, Health information exchange, Internet use, Health communication

## Abstract

**Background:**

Increased internet use for health information in the United States enhances interactions with healthcare professionals, but its effects on healthcare utilization and care quality are still being investigated. We explored the association between internet use for health information, patient-centered communication (PCC), and sociodemographic factors on the likelihood of visiting a health care provider and quality of care. We also examined if PCC mediates this association.

**Methods:**

We conducted a secondary data analysis using the National Cancer Center Institutes (NCI) Health Information National Trends Survey (HINTS) 2018–2020. Multinomial logistic regression and path analyses assessed variable interrelationships and mediating effects.

**Results:**

Individuals using the internet for health information for themselves were 2.40 times more likely (*P* <.001) to have frequent provider visits and 1.18 times more likely (*P* <.022) to rate their care as very good/good compared to excellent, compared to those who did not use the internet for health information for themselves. In contrast, individuals using the internet for discussion with their providers were 2.05 times more likely (*P* <.001) to have increased visits, and they were 40% less likely (*P* <.001) to rate their care as fair/poor compared to excellent, relative to those who did not use the internet for discussions. Path analysis indicated that individuals using the internet for health information for themselves may negatively impact PCC, resulting in lower quality ratings, while those who use the internet for discussions with healthcare providers had a positive effect on PCC, leading to higher care ratings.

**Conclusion:**

This study enhances our understanding of how PCC and internet use for health information impact US healthcare. Using the internet for provider discussions positively impacts perceived care quality, highlighting PCC’s vital role.

## Background

In the United States, there is an increasing focus on using the internet for health information, health service utilization, and care quality. People frequently utilize search engines to learn about symptoms, illnesses, and available treatments. They might read blogs, articles, and reliable health websites to educate themselves. Common search topics include diet and nutrition, exercise, certain diseases, mental health concerns, and sexual health [1].

It is critical to comprehend how the growing availability of internet use for health information affects health services and the standard of care. A sizable section of Americans—between 69.8% and 81.5%—have looked up health or medical-related information online, with 68.9% citing the internet as their primary source [2].

A literature review identified the behavioral patterns in internet use for health information seeking and its beneficial effects on health behavior. It emphasizes that a sizable majority of consumers of health information think that internet resources significantly influence their decision-making regarding their health and general maintenance of health. Furthermore, it highlights that regular use of online health resources is associated with improved adherence to medical advice [3].

The internet can affect patients’ knowledge, competence, and involvement in health decision-making strategies, which has implications for the use of health services [4]. Through the accessibility of information, people can take preventative measures to maintain their health, investigate symptoms and treatment choices, and make informed decisions about their health [5]. Furthermore, well-informed patients are more likely to ask questions, participate actively in creating individualized treatment plans, and have meaningful conversations with healthcare professionals [1, 6, 7].

Studies indicate a connection between accessing healthcare services and health information online. Recent research suggests that individuals who use healthcare services and seek health information online are more likely to engage with medical professionals [8, 9]. Furthermore, people with access to the internet are more likely to use healthcare services for early detection and preventive care, which can lead to better health outcomes [10]. Although using the internet gives patients more power, it can also create problems for the relationship between the patient and the provider. For example, patients may start to doubt medical advice after reading it online [11].

Regarding search patterns and accessibility, using smart devices, such as smartphones and tablets, for seeking health information is increasingly prevalent, with implications for medical decision-making and health behavior. People use mobile devices to look up health-related information, view medical records, and have virtual consultations with medical experts [12]. Furthermore, the use of mobile devices to search for health information is not limited to any particular demographic, as it is seen in a range of age groups and socioeconomic backgrounds [9]. The increasing prevalence of using mobile devices to look up health-related information highlights the need to comprehend how this behavior affects healthcare outcomes and decision-making.

A vital aspect affecting health service utilization and quality of care is the patient and provider relationship, commonly called patient-centered communication (PCC). PCC emphasizes a cooperative approach between healthcare providers and patients, which has become crucial in healthcare delivery. Clear communication from healthcare providers improves patients’ understanding of their health conditions and treatments [13]. In addition, studies show a strong association between effective communication and improved patient satisfaction [14].

Although there is no direct, relevant theory specifically addressing Patient-Centered Communication (PCC), the Technology Acceptance Model (TAM) helps in understanding how patients’ views of internet health information’s usefulness and ease of use affect their adoption, with PCC mediating this by personalizing support, boosting engagement, and improving healthcare utilization and quality through tailored care and trust-building [15].

Furthermore, substantial evidence suggests that PCC can act as a mediator between internet use for health information, healthcare utilization, and quality of care. Internet use improves PCC by empowering patients, resulting in informed discussions and collaborative decision-making [16–18]. This, in turn, has a positive impact on healthcare utilization and quality of care by increasing mutual understanding, patient involvement, and satisfaction levels [19–24]. Thus, PCC plays an important role in transforming internet-based health information into better healthcare outcomes.

Empirical studies further support PCC as a mediator linking access to care, e-health use, and patient experiences with improved health outcomes and healthcare quality [25–27].

This study describes the interrelationships among internet use for health information, perceived patient-centered communication (PCC), and the likelihood of visiting a health care provider and quality of care. Internet use for health information is assessed through three variables: searching for health information online, using a smart device to manage or treat a health condition, and using a smart device to have discussions with healthcare providers. These variables offer a comprehensive perspective on how individuals engage with healthcare technologies and their potential impact on healthcare utilization and quality of care.

Internet health information sources aim to educate and empower patients by offering easily available, reputable content that encourages informed decision-making, and better patient-provider communication [28, 29]. Therefore, we hypothesized that increasing internet use for health information is associated with a higher chance of healthcare visits and improved perceived quality of treatment, as mediated by patient-centered communication (PCC).

This study further assesses whether perceived patient-centered communication mediates the relationship between internet use for health information, visits to a health care provider, and quality of care.

## Methods

### Data source

Data from the study came from the National Cancer Center Institutes (NCI) Health Information National Trends Survey (HINTS) 2018 to 2020; three iterations of the nationally representative survey were designed to assess changes in health communication and how individuals use different communication channels such as the internet to obtain health information for themselves or others. Since 2003, HINTS has tracked changes in the American public use of cancer-related and health-related information. The sampling frame consisted of a database of addresses used by Marketing Systems Group (MSG) to provide random samples of addresses for national representation. HINTS employs weighted data to ensure that the results are representative of the U.S. population, accounting for nonresponse and other biases. The sample design consisted of a single-mode mail survey for respondent selection using the Next Birthday Method; reports on the conceptual framework of HINTS and other sampling designs are published on the NCI website and other sources [30].

Data for HINTS 2018 was collected from January 26 to May 2, 2018. Data for HINTS 2019 was collected from January 22nd to April 30 th, 2019. Data for HINTS 2020 was collected from February to June 2020. During a household screening, one adult (age 18 or older) was chosen from each family to participate in the survey. The response rates for each iteration are as follows: HINTS 2018 33%, HINTS 2019 30%, and HINTS 2020 37%. A final sampling weight and 50 replicate sampling weights were assigned to every sampled adult who responded. Complete interviews were conducted with 3,504 adults for HINTS 2018, 5247 adults for HINTS 2019, and 3,865 adults for HINTS 2020.

HINTS surveys use specific procedures to manage the possibility of duplicate responses from participants, such as sampling only one adult per household and using imputation techniques to combine multiple responses. Furthermore, weighting adjustments are used to ensure that the final data is representative and accurate despite any non-response or coverage errors [31].

### Measures

#### Outcomes

Respondents were asked to measure the frequency of provider visits, ‘In the past 12 months, not counting times you went to an emergency room, how many times did you go to a doctor, nurse, or other health professional to get care for yourself?’ (none, one time, two times, three times, four times, 5 to 9 times, ten or more times). Responses were recategorized into three categories (none, 1–3 times, and four or more times). To measure quality of care, respondents were asked, ‘Overall, how would you rate the quality of health care you received in the past 12 months?’ (excellent, very good, good, fair, or poor). These were recategorized as (excellent, very good/good, and fair/poor).

The decision to recategorize certain variables was made to prevent having small case numbers in specific categories while still capturing the variance of the variables and improving the interpretability of the results. We recategorized provider visit frequency into three groups—none, moderate, and high—to better reflect healthcare utilization levels. Similarly, quality of care responses were grouped to clarify patient perceptions by combining conceptually similar positive ratings while preserving distinctions from lower ones.

Additionally, this approach was intended to facilitate comparisons with previous studies in the field [32–34].

#### Independent variables

##### Internet use for health information

Three questions were used in this study. During the last 12 months (1), whether respondents have used a computer, smartphone, or other internet means to look for health or medical information for themselves (yes/no) (2) if a smartphone or tablet has helped them decide how to treat an illness or condition (yes/no) and (3) if their tablet or smartphone enabled them in discussions with their health care provider (yes/no). For brevity, we will refer to these three variables as internet use for health information for themselves, internet use for health decisions, and internet use for discussions with healthcare providers, respectively.

#### Mediator

The mediator of this study is patient-centered communication (PCC). A composite score was created by summing responses to questions about how often doctors, nurses, or other health care professionals (1) gave you the chance to ask all the health-related questions you had (2), gave the attention needed for your feelings, and emotions (3) involved you in health care decisions as much as you wanted (4), made sure that you understood the information needed to take care of your health (5) explained things in a way that you could understand (6) spent enough time with you (7) helped you deal with feelings of uncertainty. Response options were recoded so that a higher number would indicate more positive patient-centered communication (i.e., 1 = never, 2 = sometimes, 3 = usually, and 4 = always). The minimum and maximum patient-centered composite scores were 7 and 28, respectively.

This PCC scale has been evaluated for the general US adult population including measures of reliability and validity [35]. These survey questions and composite scores have been used by several other studies to measure patient-centered communication [36–38].

#### Other covariates

Age was divided into five categories: 18–34, 35–49, 50–64, 65–74, and 75+. Gender was classified as male or female. Race/ethnicity was classified as White, Black, Hispanic, or Asia. Education was assessed through four levels: less than high school, high school graduate, some college, and college graduate. Combined household income was categorized into brackets ranging from less than $20,000 to $75,000 or more. Chronic health conditions were classified as either none or one or more conditions present. Self-rated health was assessed using categories of excellent/very good, good, and fair/poor. Finally, we examined whether individuals had a regular provider they see most often (yes/no), as continuity of care influences both visit frequency and care quality. These variables were included in this study as control variables because prior studies suggested they are significantly associated with use of internet use for health information, health service utilization, and perception of quality of care [39–42].

### Analytic methods

Analyses were conducted using STATA, version 17. Descriptive analyses and bivariate analyses (chi-square test) were conducted to estimate the association between the independent variables and the outcomes, as well as the mediator and the outcomes. The study used two different sample sizes due to the nature of the survey questions. If respondents did not visit a provider in the last 12 months, they could not answer the question regarding quality of care. The provider visit frequency analysis uses the cohort (*n* = 9570). The analysis for the quality-of-care outcome was turned using the subcohort (*n* = 7,671).

The survey did not collect information on the reasons individuals chose not to seek care; however individuals from our dataset who had not sought healthcare in the last 12 months were predominantly under the age 64 (85.90%), did not have a regular provider (76.33%), did not report chronic health conditions (61.79%), and rated their health as excellent/very good (56.66%).

We used two types of analyses to assess the multivariate relationships among internet use for health information, perceived patient-centered communication quality, and the two outcomes (visiting a health care provider and quality of care). First, a series of multinomial logistic regression models were used to examine the effect of internet use for health information on the frequency of provider visits and quality of care. We then followed the suggestions from Baron and Kenny by adding the mediator into the regression models to assess whether there are significant changes in the effect of internet use for health information on outcomes to suggest PCC as a potential mediator [43].

Compared to the strength of multinomial logistic regression, which helps identify key predictors, the strength of path analysis lies in its ability to understand the interrelationships and connections among variables. Therefore, we further used path analyses to analyze whether perceived centered communication is a mediator between internet use for health information and outcomes. In addition, we calculated the direct and indirect effects to determine the extent of mediation. The path analyses were conducted using the Generalized Structural Equation Modeling (GSEM) procedure on STATA (StataCorp, 2024). A maximum-likelihood method was used as the estimator. Bootstrapping approximate model fit indexes were reported, including the Chi-squared measure, Root Mean Square Error of Approximation (RMSEA), and Comparative fit index (CFI). Initially, we included paths between all covariates and each outcome, as well as all covariates and PCC. However, the model encountered an overidentification issue. To address this, we streamlined the model by removing certain paths between the covariates and PCC and focusing solely on sociodemographic factors and the self-rated health variable. Bootstrapped confidence intervals (10,000 resamples) were used to ensure robust mediation estimates. All the analyses were conducted separately by outcome. Statistical significance was set at alpha = 0.05 in all analyses.

## Results

### Sample characteristics and bivariate analyses

Table 1 displays the sample characteristics of internet users in the HINTS surveys. In both cohorts, most participants were non-Hispanic white, had a combined household income over $50,000, and had an education level of some college or above. Most participants also had one or more chronic health conditions but indicated a very good or excellent self-rated health score. In addition, most participants had a regular healthcare provider. Most participants also indicated that they had used the internet for health information themselves in the last 12 months, had excellent or very good quality of care, and had positive experiences with patient-centered communication.

**Table 1:** Characteristics of the study sample

Variables	Cohort (*N*= 9,570)	Sub cohort (*N*=7,671)
	**n (%)**	**n (%)**
Gender		
Female	5,593 (58.44)	4,618 (60.20)
Male	3,977 (41.56)	3,053 (39.80)
Race		
Non-Hispanic White	5,769 (60.28)	4,784 (62.36)
Non-Hispanic Black or African American	1,160 (12.12)	923 (12.03)
Hispanic	1,379 (14.41)	1,005 (13.10)
Non-Hispanic Asian	450 (4.70)	317 (4.13)
Non-Hispanic Others and Missing Data	812 (8.48)	642 (8.37)
Age		
18–34	1,468 (15.34)	1,123 (14.64)
35–49	2,124 (22.19)	1,639 (21.37)
50–64	3,183 (33.26)	2,560 (33.37)
65–74	1,964 (20.52)	1,650 (21.51)
75+	831 (8.68)	699 (9.11)
Household Income)		
Less than $20,000	1,180 (12.33)	891 (11.62)
$20,000 to < $35,000	1,012 (10.57)	783 (10.21)
$35,000 to < $50,000	1,156 (12.08)	900 (11.73)
$50,000 to < $75,000	1,696 (17.72)	1,357 (17.69)
$75,000 or More	3,855 (40.28)	3,212 (41.87)
Missing Data	671 (7.01)	528 (6.88)
Educational Level		
Less than High School	430 (4.49)	301 (3.92)
High School Graduate	1,407 (14.70)	1,056 (13.77)
Some College	2,858 (29.86)	2,280 (29.72)
College Graduate or More	4,875 (50.94)	4,034 (52.59)
Chronic health conditions		
None	3,646 (38.10)	2,668 (34.78)
One or more	5,924 (61.90)	5,003 (65.22)
In general, would you say your health is…		
Excellent/very good	5,000 (52.25)	3,956 (51.57)
Good	3,322 (34.71)	2,695 (35.13)
Fair/poor	1,248 (13.04)	1,020 (13.30)
Regular Provider		
Yes	6,738 (70.41)	5,929 (77.29)
No	2,832 (29.59)	1,742 (22.71)
Frequency of Visiting Provider Visit		
None	1,149 (12.01)	–^a^
1–3 times	4,768 (49.82)	4,267 (55.63)
4 or more times	3,653 (38.17)	3,404 (44.37)
Internet Use for Health Information for Themselves		
Yes	7,572 (79.12)	6,281 (81.88)
No	1,998 (20.88)	1,390 (18.12)
Internet Use for Healthcare Decisions		
Yes	3,925 (41.01)	3,265 (42.56)
No	5,645 (58.99)	4,406 (57.44)
Internet Use for Discussions with Healthcare Providers		
Yes	3,768 (39.37)	3,247 (42.33)
No	5,802 (60.63)	4,424 (57.67)
Quality of Care, mean (SD)	–	4.04^b^ (0.88^c^)
Quality of Care		
Excellent	–	2,625 (34.22)
Very Good	–	3,177 (41.42)
Good	–	1,473 (19.20)
Fair	–	329 (4.29)
Poor	–	67 (0.87)
Patient-Centered Communication, mean (SD)	–	23.95^b^ (4.36^c^)

^a^ Not Applicable

^b^ Mean score

^c^ Standard deviation of mean score

Bivariate associations between study variables, frequency of provider visits, and quality of care are shown in Table 2. Respondents who had a higher frequency of healthcare provider visits (more than 4 times) were female (40.89%), non-Hispanic white (40.28%), and in the older age groups (54.39%). Those who had a higher frequency of 1–3 visits had higher household combined income (53.64%), higher education levels (51.82%), had a regular provider (49.57%), had excellent or very good self-rated health (57.32%), and used all three forms of the internet for health purposes (49.46%, 48.08%, and 47.61%). Respondents with higher ratings for quality-of-care (very good) were non-Hispanic white (41.77%), in the older age groups (40.49%), in the higher household incomes (44.08%), had higher education levels (43.21%), had a regular provider (41.88%), and used their smartphone to discuss with their healthcare provider (40.93%). Those who had higher frequencies of rating their care as excellent had high scores for patient-centered communication (55.03%).

**Table 2:** Bivariate analysis of the study sample

Variable	Frequency of Provider Visit *n* = 9,570 (%)				Quality of Care *n* = 7,671 (%)				
	None	1–3	≥ 4	*P *value	Excellent	Very Good	Good	Fair/Poor	*P *value
Gender									
Female	10.08	49.03	40.89	< 0.001	34.78	41.17	19.06	5.00	0.586
Male	14.71	50.94	34.35		33.38	41.79	19.42	5.40	
Race									
Non-Hispanic White	9.88	49.84	40.28	< 0.001	37.21	41.70	16.99	4.10	< 0.001
Non-Hispanic Black or African American	11.38	49.05	39.57		29.47	44.75	19.83	5.96	
Hispanic	19.58	50.54	29.88		30.25	37.41	24.58	7.76	
Non-Hispanic Asian	18.89	52.00	29.11		20.82	44.16	27.13	7.89	
Non-Hispanic Others and Missing Data	11.33	48.40	40.27		31.62	39.41	22.43	6.54	
Age									
18–34	18.32	50.34	31.34	< 0.001	29.39	37.22	24.49	8.90	< 0.001
35–49	16.71	52.45	30.84		31.30	41.00	21.72	5.98	
50–64	11.40	50.39	38.20		33.44	43.55	18.24	4.77	
65–74	6.72	48.98	44.30		39.52	41.76	15.64	3.09	
75+	3.61	42.00	54.39		39.20	40.49	16.74	3.58	
Combined Household Income									
Less than $20,000	16.19	38.90	44.92	< 0.001	30.75	34.12	23.57	11.56	< 0.001
$20,000 to < $35,000	14.43	48.81	36.76		33.33	37.68	22.48	6.51	
$35,000 to < $50,000	13.49	51.04	35.47		33.33	43.00	18.89	4.78	
$50,000 to < $75,000	12.56	47.70	39.74		33.68	40.46	20.34	5.53	
$75,000 or More	9.68	53.64	36.68		35.99	44.08	16.84	3.08	
Missing Data	10.43	51.86	37.70		33.52	42.80	18.94	4.73	
Educational Level									
Less than High School	21.16	42.56	36.28	0.046	31.23	33.55	24.92	10.30	< 0.001
High School Graduate	16.20	48.83	34.97		32.72	41.38	20.74	6.16	
Some College	12.67	48.01	39.33		34.21	39.30	20.83	39.30	
College Graduate or More	9.60	51.82	38.58		35.10	43.21	17.45	4.24	
Chronic health conditions									
None	19.47	55.49	25.04	< 0.001	33.88	42.28	18.89	4.95	0.695
One or more	7.41	46.34	46.25		34.40	40.96	19.37	5.28	
In general, would you say your health is…									
Excellent/very good	13.02	57.32	29.66	< 0.001	42.06	42.57	12.87	2.50	< 0.001
Good	11.26	45.67	43.08		26.42	41.86	26.57	5.16	
Fair/poor	9.94	30.85	59.21		24.41	35.78	24.31	15.49	
Regular Provider									
Yes	4.04	49.57	46.39	< 0.001	37.14	41.88	17.00	3.98	< 0.001
No	30.97	50.42	18.61		24.28	39.84	26.69	9.18	
Internet Use for Health Information for Themselves									
Yes	9.93	49.46	40.61	< 0.001	33.66	41.78	19.34	5.22	0.179
No	19.87	51.20	28.93		36.76	39.78	18.56	4.89	
Internet Use for Healthcare Decisions									
Yes	10.29	48.08	41.63	< 0.001	33.29	40.95	20.03	5.73	0.064
No	13.20	51.04	35.77		34.91	41.76	18.59	4.74	
Internet Use for Discussions with Healthcare Providers									
Yes	6.82	47.61	45.57	< 0.001	36.37	40.93	18.29	4.40	< 0.001
No	15.37	51.26	33.37		32.64	41.77	19.87	5.72	
Patient-Centered Communication									
Low (< 25 th percentile)	–	–	–		7.62	37.65	40.56	14.16	< 0.001
Moderate (25 th −50 th percentile)	–	–	–		23.38	56.40	17.33	2.88	
High (> = 50 th percentile)	–	–	–		55.03	36.84	7.30	0.83	

### Multinomial regression analysis

The results of the multinomial regression analysis for frequent provider visits are presented in Table 3. In Model 1a and 1b, respondents who used the internet to search for health information for themselves are 1.67 times more likely to have 1–3 visits (RRR = 1.67, 95% CI 1.40–1.98; *P* <.001) and 2.40 times more likely to have 4 or more visits (RRR = 2.40, 95% CI: 1.97–2.91; *P* <.001), compared to no visits. Respondents who used the internet to help them with discussions with healthcare providers are 1.55 times more likely to have 1–3 visits (RRR = 1.55, 95% CI: 1.29–1.85; *P* <.001) and 2.05 times more likely to have 4 or more visits (RRR = 2.05, 95% CI 1.69–2.47; *P* <.001), compared to no visits. When adding the mediator in Model 2a and Model 2b, the significant effect of using the internet to search for health information for themselves and using the internet to help with discussions with health care providers remained, indicating patient-centered communication may not be the mediator in these relationships.

**Table 3:** Logistic regression analysis of internet use for health information and provider visits (*n* = 9,570)^a^

	Model 1a: 1–3 Visits vs. None		Model 1b: ≥ 4 Visits vs. None		Model 2a: 1–3 Visits vs. None + mediator		Model 2b: ≥4 Visits vs. None + mediator	
	RRR^b^ (95% CI)	*P* value	RRR (95% CI)	*P* value	RRR (95% CI)	*P* value	RRR (95% CI)	*P* value
Internet Use for Health Information for Themselves (ref. No)								
Yes	1.67 (1.40–1.98)	< 0.001	2.40 (1.97–2.91)	< 0.001	1.58 (1.32–1.90)	< 0.001	2.25 (1.84–2.74)	< 0.001
Internet Use for Healthcare Decisions (ref. No)								
Yes	0.89 (0.76–1.06)	0.195	0.95 (0.79–1.13)	0.553	0.88 (0.74–1.04)	0.142	0.92 (0.76–1.12)	0.392
Internet Use for Discussions with Healthcare Providers (ref. No)								
Yes	1.55 (1.29–1.85)	< 0.001	2.05 (1.69–2.47)	< 0.001	1.54 (1.29–1.85)	< 0.001	2.04 (1.68–2.48)	< 0.001
Patient-Centered Communication (cont.)								
	–		–		1.02 (1.02–1.03)	< 0.001	1.03 (1.03–1.04)	< 0.001

^a^Covariates in this analysis include age, gender, race/ethnicity, education, combined household income, chronic health conditions, self-rated health, and regular provider

^b^Relative Risk Ratio

Table 4 presents the results of the multinomial regression analysis for quality of care. Respondents who have used the internet to search for health information for themselves are 1.18 times more likely to rate their quality of care as very good/good compared to excellent (RRR = 1.18, 95% CI: 1.02–1.35; *P* =.022). Respondents who used the internet for health decisions are 1.39 times more likely to rate their quality of care as fair/poor compared to excellent (RRR = 1.39, 95% CI 1.08–1.80; *P* =.011). However, respondents who used the internet to have discussions with their healthcare provider are 21% less likely to rate their care as very good/good (RRR = 0.79, 95% CI 0.70–0.89; *P* <.001) and 40% less likely to rate their care as fair/poor (RRR = 0.60, 95% CI 0.46–0.78; *P* <.001), compared to excellent. When adding the mediator, the effect of internet use for health information for themselves disappeared, indicating a potential mediating effect of patient-centered communication on this relationship. The IRR for internet use for discussions with health care providers changed from 0.60 to 0.65 for fair/poor, indicating a partial mediating effect.

**Table 4:** Logistic regression analysis of internet use for health information and quality of care (*N* = 7,671)^a^

	Model 1a: Very Good/Good vs. Excellent		Model 1b: Fair/Poor vs. Excellent		Model 2a: Very Good/Good vs. Excellent + mediator		Model 2b: Fair/Poor vs. Excellent + mediator	
	RRR (95% CI)	*P* value	RRR (95% CI)	*P* value	RRR (95% CI)	*P* value	RRR (95% CI)	*P* value
Internet Use for Health Information for Themselves (ref. No)								
Yes	1.18 (1.02–1.35)	0.022	1.25 (0.90–1.72)	0.169	1.06 (0.91–1.24)	0.415	1.03 (0.71–1.49)	0.875
Internet Use for Healthcare Decisions (ref. No)								
Yes	1.10 (0.98–1.24)	0.091	1.39 (1.08–1.80)	0.011	1.09 (0.96–1.24)	0.163	1.49 (1.11–1.99)	0.008
Internet Use for Discussions with Healthcare Providers (ref. No)								
Yes	0.79 (0.70–0.89)	< 0.001	0.60 (0.46–0.78)	< 0.001	0.79 (0.70–0.90)	< 0.001	0.65 (0.48–0.88)	0.005
Patient-Centered Communication (cont.)								
	–		–		0.72 (0.71–0.74)	< 0.001	0.56 (0.54–0.58)	< 0.001

^a^Covariates in this analysis include age, gender, race/ethnicity, education, combined household income, chronic health conditions, self-rated health, and regular provider

### Path analyses and the direct and indirect effects

**Fig. 1:**
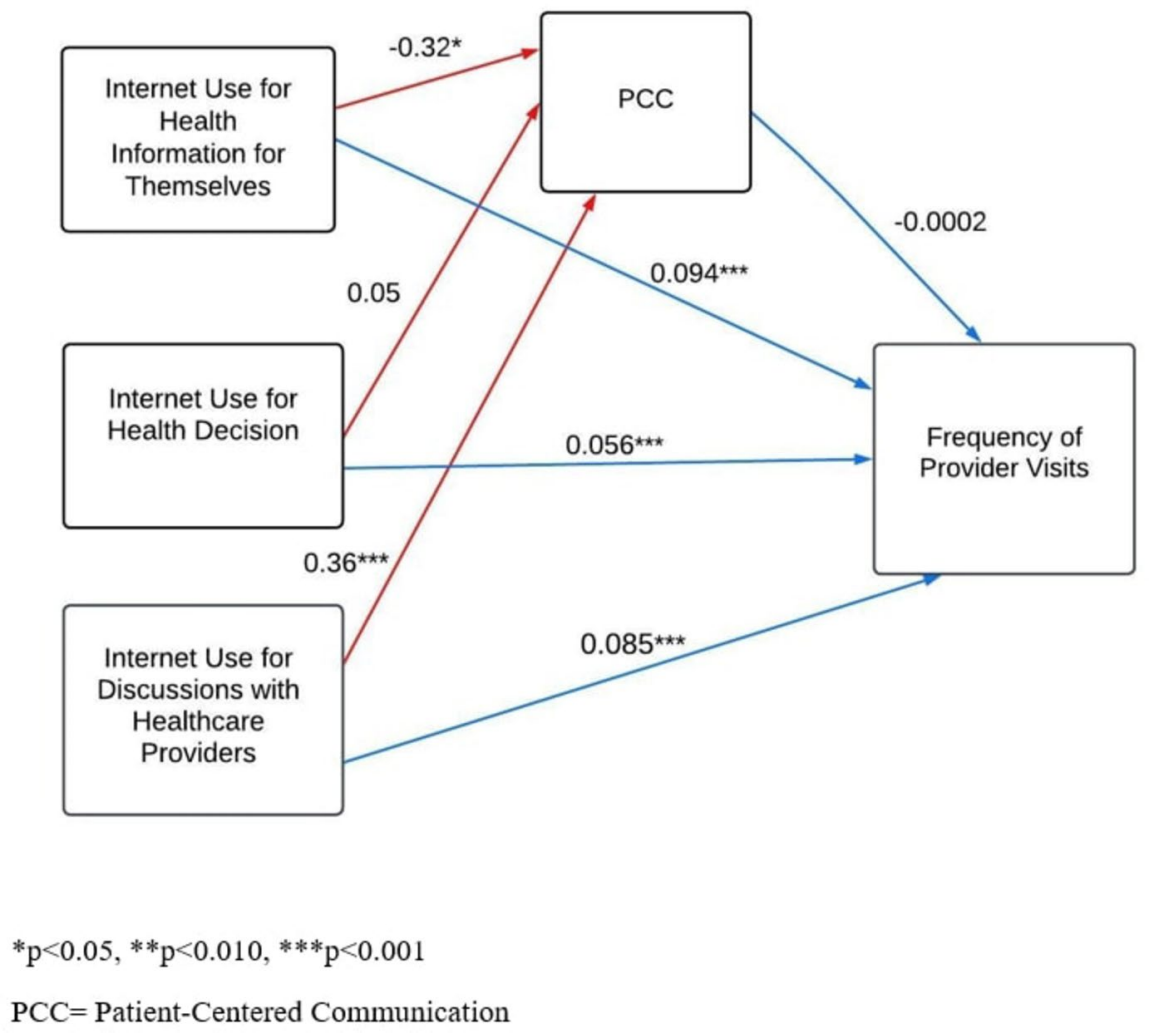
Path analysis of internet use for health information, PCC, and Frequency of Provider Visits

**Fig. 2:**
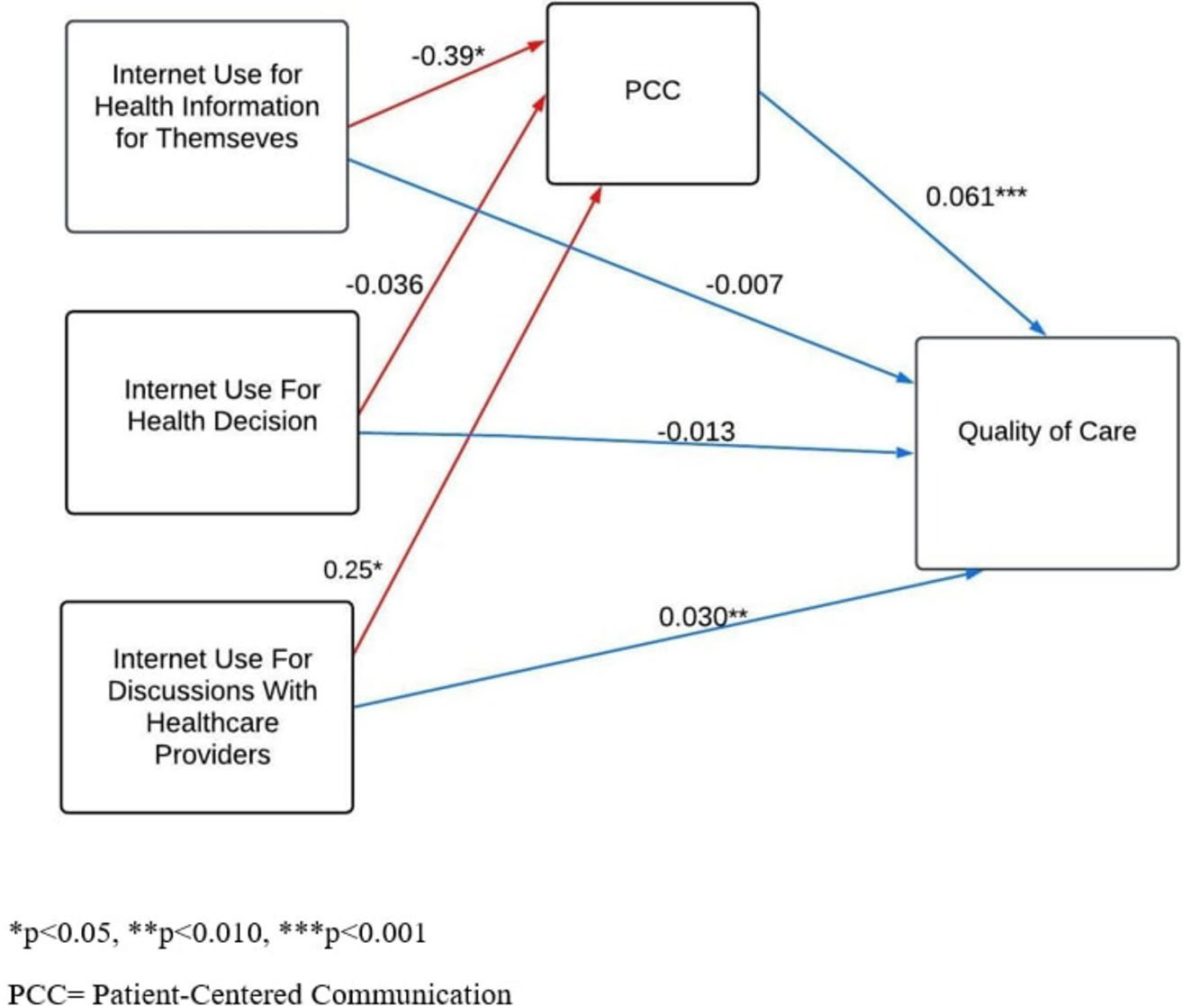
Path analysis of internet use for health information, PCC, and Quality of Care

Figures 1 and 2 present the results of the path analyses for the frequency of provider visits and quality of care respectively. The decomposition of the direct and indirect effects was reported in Table 5.

**Table 5:** Direct and indirect effects of internet use for health information on outcomes

Predictor and Path	Total Effects		Direct Effects		Indirect Effects	
	β (95% CI)	*P* value	β (95% CI)	*P* value	β (95% CI)	*P* value
Predictor on Frequency of Provider Visits (FPV)						
Internet Use for Health Information for Themselves →FPV	0.094 (0.07–0.12)	< 0.001	0.094 (0.06–0.12)	< 0.001	0.00006 (−0.0009–0.001)	0.888
Internet Use for Health Decisions →FPV	0.056 (0.03–0.07)	< 0.001	0.056 (0.03–0.07)	< 0.001	0.00001 (−0.0001-0.0001)	0.806
Internet Use for Discussions with Healthcare Providers →FPV	0.085 (0.06–0.11)	< 0.001	0.085 (0.06–0.12)	< 0.001	−0.0001 (−0.001-0.0006)	0.576
PCC →FPV	-		−0.0002 (−0.002-0.002)	0.888	-	
Predictor on Quality of Care (QC)						
Internet Use for Health Information for Themselves → QC	−0.030 (−0.06-0.002)	0.068	−0.007 (−0.04-0.02)	0.633	−0.023 (−0.04 - −0.007)	0.004
Internet Use for Health Decisions → QC	−0.014 (−0.04-0.009)	0.233	−0.013 (−0.04-0.008)	0.246	−0.002 (−0.01-0.009)	0.716
Internet Use for Discussions with Healthcare Providers → QC	0.046 (0.02–0.07)	< 0.001	0.030 (0.008–0.05)	0.006	0.015 (0.003–0.03)	0.012
PCC →QC	-		0.061 (0.05–0.06)	< 0.001	-	

In our path analysis (Fig. 1), we examined the relationships between internet use for health information variables, PCC, and frequency of provider visits. The model revealed significant positive paths from internet use for health information for themselves (β = 0.094, *P* <.001), internet use for health decisions (β = 0.056, *P* <.001), and internet use for discussions with healthcare providers (β = 0.085, *P* <.001) to frequency of provider visits indicating that higher use of internet for health purposes is associated with higher frequencies of provider visits. Furthermore, internet use for health information for themselves had a significant negative path (β = −0.33, *P* <.05) to PCC while internet use for discussions with healthcare providers had a significant positive path (β = 0.36, *P* <.001) to PCC. This suggests that lower use of the internet for health information for themselves is associated with higher ratings of PCC, while higher use of the internet for discussions with healthcare providers is associated with high ratings of PCC. Regarding the model fit indices, although the Chi-square test was significant, it is sensitive to sample size. Therefore, additional fit indices such as CFI and RMSEA provide a more comprehensive evaluation of the model’s fit to the data. The results indicated that the model fit was acceptable (Chi-squared [18] = 1195, CFI = 0.922, RMSEA = 0.078).

In Table 5, For frequency of provider visits, the significant direct effects (β = 0.094, β = 0.056, β = 0.085, *P* <.001 respectively) and insignificant indirect effects for all three dimensions of internet use for health information suggested these internet-using behaviors for health information do not exert their influence on frequency provider visit via patient-centered communication. They either influence the frequency of provider visits directly or through other unmeasured variables. The insignificant direct effect of patient-centered communication on provider visit frequency double-confirmed that patient-centered communication is not the mediator.

In Fig. 2, we examined the relationships between internet use for health information variables, PCC, and quality of care. The model revealed a significant positive path from internet use for discussions with healthcare providers (β = 0.030, *P* <.001) to quality of care, indicating that higher use of the internet for discussions with healthcare providers is associated with higher quality of care. Furthermore, internet use for health information for themselves had a significant negative path (β = −0.39, *P* <.05) to PCC while internet use for discussions with healthcare providers had a significant positive path (β = 0.25, *P* <.05) to PCC. This suggests that lower use of the internet for health information for themselves is associated with higher ratings of PCC, while higher use of the internet for discussions with healthcare providers is associated with high ratings of PCC. In addition, there was a significant positive path from PCC (β = 0.061, *P* <.001) to quality of care, indicating that higher PCC is associated with higher quality of care. Model fit statistics indicated good fit (Chi-squared [18] = 3026, CFI = 0.969, RMSEA = 0.078).

In Table 5, regarding the quality of care, the direct effect of internet use for health information for themselves on the quality of care is insignificant. However, the indirect effect is significant (β = −0.023, 95% CI: −0.04 - −0.007, *P* =.004), indicating that internet use for health information influences the quality of care through patient-centered visit communication. Regarding internet use for discussion with health care providers, direct (β = 0.030, 95% CI:0.008–0.05, *P* =.006) and indirect effects (β = 0.015, 95% CI:0.003–0.03, *P* =.012) are significant. The total effects reached a value of 0.046 (95% CI:0.02–0.07, *P* <.001) with statistical significance. The direct effects of patient-centered communication on the quality of care are also significant, with a value of 0.061 (95% CI: 0.05–0.06, *P* <.001). The above results suggested that patient-centered communication partially mediates the relationship between internet use for discussion with healthcare providers and quality of care.

## Discussion

This study yields valuable insights into the relationships among internet use for health information, patient-centered communication (PCC), healthcare utilization, and quality of care within a nationally representative sample of American adults. To the best of our knowledge, this study is the first to explore the association of internet use for health information with quality of care and health service utilization, with PCC acting as a mediator at the population level.

The multinomial logistic regression analysis revealed positive effects for both internet use for health information for themselves and internet use for discussions with healthcare providers on the frequency of provider visits. According to previous studies, individuals who communicated with medical professionals using the internet were more likely to say that their smartphone or tablet assisted them in making medical decisions. This shows that patients’ empowerment and engagement in their healthcare journey can be improved by mobile devices [12, 44].

Further analysis regarding the quality of care revealed interesting connections. When including PCC in the multinomial regression, internet use for health information for themselves was not significant, suggesting a mediation effect. This could be due to several factors, such as how the respondents use or interpret information they found on the internet during their healthcare visit and their relationship with their healthcare provider [45]. On the other hand, the direction of the relationship between internet use for health information for themselves and quality of care may be reversed, meaning that poorer quality of care experiences lead to a higher likelihood of searching for health information online [46]. In contrast, internet use for discussions remained significant when adding PCC into the multinomial regression, albeit with an increased RRR. This indicated that PCC does not mediate this relationship, suggesting the influence of other variables. Furthermore, internet use for discussions with healthcare providers remains significant when adding PCC into the multinomial regression, indicating a partial mediation. Notably, internet use for discussions with healthcare providers was significantly associated with higher ratings of quality of care, presenting a different directional relationship compared to the other variables.

To better understand the mediating role of PCC, the path analysis revealed some complex findings. Those using the internet for health information for themselves may negatively impact PCC, which in turn contributes to lower ratings of quality of care. The path analysis findings are consistent with previous studies, highlighting the potential drawbacks of internet use for health information regarding interactions with healthcare providers and healthcare ratings [47, 48]. Conflicts in the patient-physician relationship may arise from patients’ ability to question or undermine the authority of medical experts and excessive use of health information can cause worry and uncertainty [4, 49]. If these concerns are properly addressed, they may lead to a positive experience between patients and their healthcare providers.

The path analysis for quality of care also indicated significant and positive effects of internet use for discussions with healthcare providers and PCC. This highlights the potential benefits of using internet health information. As individuals become more engaged in their health care through online resources, they can foster better relationships with their providers and therefore have better quality interactions during their healthcare visits. Compared to previous studies, these results similarly demonstrated that individuals using eHealth to communicate with healthcare providers can increase the perceived quality of their care [50, 36].

This study contributes significantly to our comprehension of how the internet is used for health information and its tangible effects on real-world health outcomes. While internet use for health information can positively affect the perceived quality of care when it’s used to facilitate discussions with providers, merely using it to seek health information or make personal health care decisions may not result in the same benefits. Nevertheless, leveraging online resources to foster patient-centered discussions is an important aspect associated with patient-centered communication, which is key to quality of care.

### Limitations

Although this study resulted in several key findings, it also had several limitations. HINTS is a cross-sectional survey that prevents any assumption of causality between internet use for health information, health service utilization, and quality of care. Furthermore, HINTS respondents are primarily white, high-income, and more educated. Therefore, this sample may not be representative of the US population. Future studies should focus on populations less likely to utilize healthcare resources and lower quality of care ratings, such as Hispanics and African Americans [51, 52]. In addition, the survey questions used in this study referred to experiences in the past 12 months, which may affect the respondent’s ability to recall the information accurately.

Future studies could explore additional mediators, such as e-health literacy and trust in healthcare providers. The ability to effectively seek and apply online information can enhance patient interaction and compliance [53]. Also, patients who trust their providers are more likely to discuss online health information, leading to better communication and shared decision making [45, 54]. These mediators could not be examined in this study due to the absence of relevant measures in the HINTS dataset.

## Conclusion

Although our cross-sectional study design limits the ability to make causal inferences about the relationships between variables, our findings indicate that individuals who use the internet for health information for themselves and for discussions with healthcare providers tend to have more frequent visits. However, specific dimensions of internet use, primarily internet use for discussions with healthcare providers, positively affect the quality of care through PCC.

These findings highlight the potential of digital tools to improve patient outcomes and suggest that future research should focus on the specific mechanisms by which internet use for health information influences healthcare utilization and quality of care. Furthermore, the findings highlight the importance of promoting digital health literacy among patients so that they can effectively navigate online information and make informed health decisions. Policymakers should take these findings into account when developing guidelines for the dissemination of online health information, ensuring that all individuals have access to accurate and reliable resources.

Overall, our findings highlight the transformative power of digital health tools in improving patient outcomes and call for additional research into the mechanisms by which internet use influences healthcare behaviors and experiences across diverse populations. This will inform future strategies aimed at optimizing healthcare delivery in an increasingly digital age.

## Data Availability

The datasets analyzed during the current study are available in the National Cancer Institute (NCI) Health Information National Trends Survey (HINTS) repository, https://hints.cancer.gov/.

## Abbreviations

PCC: Patient-Centered Communication
HINTS: Health Information National Trends Survey
